# A systematic review of the therapeutic effects of resveratrol in combination with 5-fluorouracil during colorectal cancer treatment: with a special focus on the oxidant, apoptotic, and anti-inflammatory activities

**DOI:** 10.1186/s12935-022-02561-7

**Published:** 2022-04-02

**Authors:** Hossein Moutabian, Mehrsa Majdaeen, Ruhollah Ghahramani-Asl, Masoumeh Yadollahi, Esmaeil Gharepapagh, Gholamreza Ataei, Zahra Falahatpour, Hamed Bagheri, Bagher Farhood

**Affiliations:** 1 Radiation Sciences Research Center (RSRC), AJA University of Medical Sciences, Tehran, Iran; 2 Department of Radiotherapy and Oncology, Razi Hospital, Guilan University of Medical Sciences, Rasht, Iran; 3 Department of Medical Physics and Radiological Sciences, Faculty of Paramedicine, Sabzevar University of Medical Sciences, Sabzevar, Iran; 4 Department of Allied Medical Sciences, Semnan University of Medical Sciences, Semnan, Iran; 5 Medical Radiation Sciences Research Team, Tabriz University of Medical Science, Tabriz, Iran; 6 Department of Radiology Technology, Faculty of Paramedical Sciences, Babol University of Medical Sciences, Babol, Iran; 7 Department of Medical Physics, Tehran University of Medical Sciences, Tehran, Iran; 8 Radiation Biology Research Center, Iran University of Medical Sciences, Tehran, Iran; 9 Trauma Research Center, Kashan University of Medical Sciences, Kashan, Iran; 10 Department of Medical Physics and Radiology, Faculty of Paramedical Sciences, Kashan University of Medical Sciences, Kashan, Iran

**Keywords:** Colorectal cancer, Chemotherapy, 5-fluorouracil, Resveratrol, Systemic review

## Abstract

**Purpose:**

5-fluorouracil (5-FU), an effective chemotherapy drug, is commonly applied for colorectal cancer treatment. Nevertheless, its toxicity to normal tissues and the development of tumor resistance are the main obstacles to successful cancer chemotherapy and hence, its clinical application is limited. The use of resveratrol can increase 5-FU-induced cytotoxicity and mitigate the unwanted adverse effects. This study aimed to review the potential therapeutic effects of resveratrol in combination with 5-FU against colorectal cancer.

**Methods:**

According to the PRISMA guideline, a comprehensive systematic search was carried out for the identification of relevant literature in four electronic databases of PubMed, Web of Science, Embase, and Scopus up to May 2021 using a pre-defined set of keywords in their titles and abstracts. We screened 282 studies in accordance with our inclusion and exclusion criteria. Thirteen articles were finally included in this systematic review.

**Results:**

The in vitro findings showed that proliferation inhibition of colorectal cancer cells in the groups treated by 5-FU was remarkably higher than the untreated groups and the co-administration of resveratrol remarkably increased cytotoxicity induced by 5-FU. The in vivo results demonstrated a decrease in tumor growth of mice treated by 5-FU than the untreated group and a dramatic decrease was observed following combined treatment of resveratrol and 5-FU. It was also found that 5-FU alone and combined with resveratrol could regulate the cell cycle profile of colorectal cancer cells. Moreover, this chemotherapeutic agent induced the biochemical and histopathological changes in the cancerous cells/tissues and these alterations were synergized by resveratrol co-administration (for most of the cases), except for the inflammatory mediators.

**Conclusion:**

The results obtained from this systematic review demonstrated that co-administration of resveratrol could sensitize the colorectal cancer cells to 5-FU treatment via various mechanisms, including regulation of cell cycle distribution, oxidant, apoptosis, anti-inflammatory effects.

## Introduction

Colorectal cancer is the third and second most frequently diagnosed cancer among males and females worldwide, respectively [1]. Furthermore, it is the second most lethal malignancy worldwide [2]. Colorectal cancer is affected by both different genetic and environmental factors, each to a different degree in various patients [3]. The choice of first-line treatment for colorectal cancer patients currently comprises a multimodal approach based on cancer-related features and patient-related factors [4].

One of the therapeutic modalities for colorectal cancer is using different chemotherapy regimens; especially, it is considered as the mainstay of treatment in metastatic colorectal cancer. There are various chemotherapeutic drugs for managing colorectal cancer patients, including 5-fluorouracil (5-FU), capecitabine, oxaliplatin, cetuximab, irinotecan, bevacizumab, etc. [5, 6]. 5-FU is a common and conventional treatment for colorectal cancer which is used for five decades. Nevertheless, its toxicity to normal tissues and appearance of colorectal cancer chemoresistance are main obstacles to successful cancer chemotherapy and hence, its clinical application is limited [7, 8]. Therefore, identification of new therapeutic agents for their potential application combined with 5-FU during colorectal cancer treatment is warranted to improve patient survival and alleviate adverse effects.

**Fig. 1:**
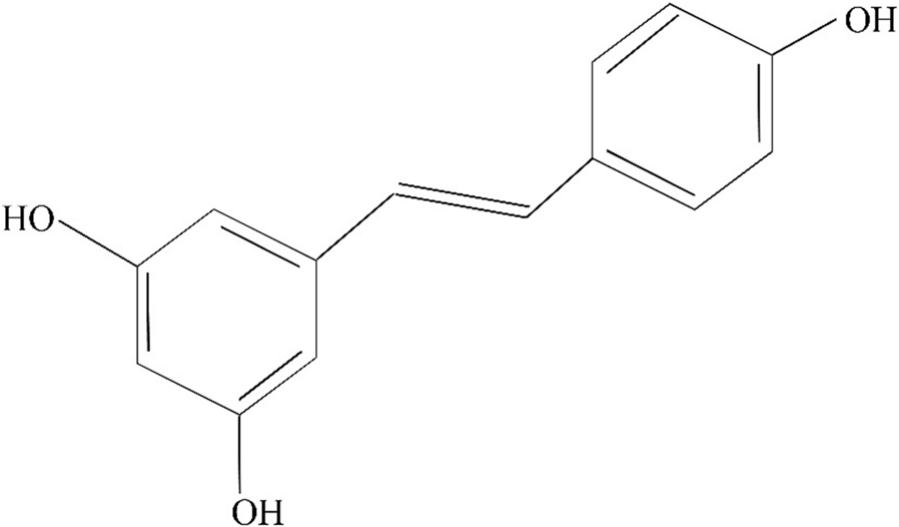
Chemical structure of resveratrol

The tendency to use herbal and natural products or their derivatives to mitigate the chemotherapy-induced side effects (chemo-protectors) or enhance the sensitivity of tumoral cells to chemotherapeutic agents (chemo-sensitizers) has attracted much attention during the past several decades. Resveratrol (3,5,4′-trihydroxy-trans-stilbene, Fig. 1) is a natural polyphenol which has been found in more than 70 plant species and its main sources are grapes, soy and peanuts [9–13]. Resveratrol is naturally able to protect herbs against fungal, ultra-violet rays, and other stresses [14]. It is also known that this herbal agent has potent anti-oxidant and anti-clastogenic properties, helping to protect against carcinogenesis and genomic instability [15, 16]. Moreover, its anti-cancer activity has been investigated in many cancer types, such as colorectal, prostate, lung, liver, breast cancers, and so on [17–21]. It has been also reported that resveratrol not only acts a chemotherapeutic agent, but also have chemo-preventive properties which are related to its anti-inflammatory, anti-oxidant, anti-apoptosis, and anti-proliferative activities [22–24]. Indeed, resveratrol targets some components of intracellular signaling pathways such as tumor angiogenic and metastatic switches, pro-inflammatory mediators, and cell survival and apoptosis regulators by modulating a distinct set of transcription factors (including p53, aryl hydrocarbon receptor, nuclear factor-erythroid 2-related factor 2 (Nrf2), forkhead box subgroup O, activating transcription factor 3, nuclear factor kappa B (NF-κB), etc.), upstream kinases (including mitogen-activated protein kinases (MAPKs), protein kinase C, and Akt, etc.) and their regulators (including early growth response-1, Krüppel-like factor-4, IκBα kinase (IKK), cyclooxygenase (COX)‐2, vascular endothelial growth factor (VEGF), Bcl2, matrix metalloproteinase (MMP)-9, etc.) [25–29].

In the current study, a systematic search was carried out on the potential therapeutic role of resveratrol during colorectal cancer chemotherapy by 5-FU. Additionally, we tried to answer the following issues: (1) the underlying mechanisms of toxicities induced by 5-FU chemotherapeutic agent on colorectal cancer cells, (2) the role of resveratrol on 5-FU‐induced toxicities on colorectal cancer cells, and 3) the underlying mechanisms related to the chemo-sensitization role of resveratrol on colorectal cancer cells during 5-FU chemotherapy.

## Methods

### Search strategy

According to the Preferred Reporting Items for Systematic Reviews and Meta-Analyses (PRISMA) guideline [30], we performed a systematic search to assess all relevant studies on “the effects of resveratrol in combination with 5-FU against colorectal cancer” in both medical subject heading (MeSH) or advance at four electronic databases including Web of Science, Embase, PubMed, and Scopus up to May 2021 using the keywords “Resveratrol” AND “Chemotherapy” OR “5-Fluorouracil” OR “5Fluorouracil” OR “5-FU” OR “5FU” OR “Fluorouracil” OR “Adrucil” OR “Carac” AND “Colorectal cancer” OR “Bowel cancer” OR “Colon cancer” OR “Rectal cancer” OR “Rectum cancer” OR “Colorectal malignancy” OR “Bowel malignancy” OR “Colon malignancy” OR “Rectal malignancy” OR “Rectum malignancy” OR “Colorectal neoplasm” OR “Bowel neoplasm” OR “Colon neoplasm” OR “Rectal neoplasm” OR “Rectum neoplasm” OR “Colorectal carcinoma” OR “Bowel carcinoma” OR “Colon carcinoma” OR “Rectal carcinoma” OR “Rectum carcinoma” OR “Colorectal tumor” OR “Bowel tumor” OR “Colon tumor” OR “Rectal tumor” OR “Rectum tumor” in keywords, titles or abstracts.

### Study selection

The inclusion criteria considered for this systematic review were full-text articles with (1) English language, (2) our per-defined purpose on the role of resveratrol during colorectal cancer chemotherapy by 5-FU (based on the aforementioned keywords), (3) sufficient information, (4) no restriction on publication year, and (5) no restriction in publications with clinical, in-vivo, or in-vitro studies. For exclusion criteria, we excluded (1) not related papers, (2) review articles, (3) oral presentations, (4) posters, (5) case reports, (6) editorials, (7) letters to the editors, and (8) book chapters.

### Data extraction

Each eligible study was reviewed by two researchers and the following data were then extracted: (a) author name and year of publication, (b) models (clinical, in-vivo or/and in-vitro), (c), 5-FU dosage and route of administration type, (d) outcomes of colorectal cancer chemotherapy, (e) resveratrol dosage and route of administration type, and (f) resveratrol co-administration outcomes.

## Results

### Literature search and screening

**Fig. 2:**
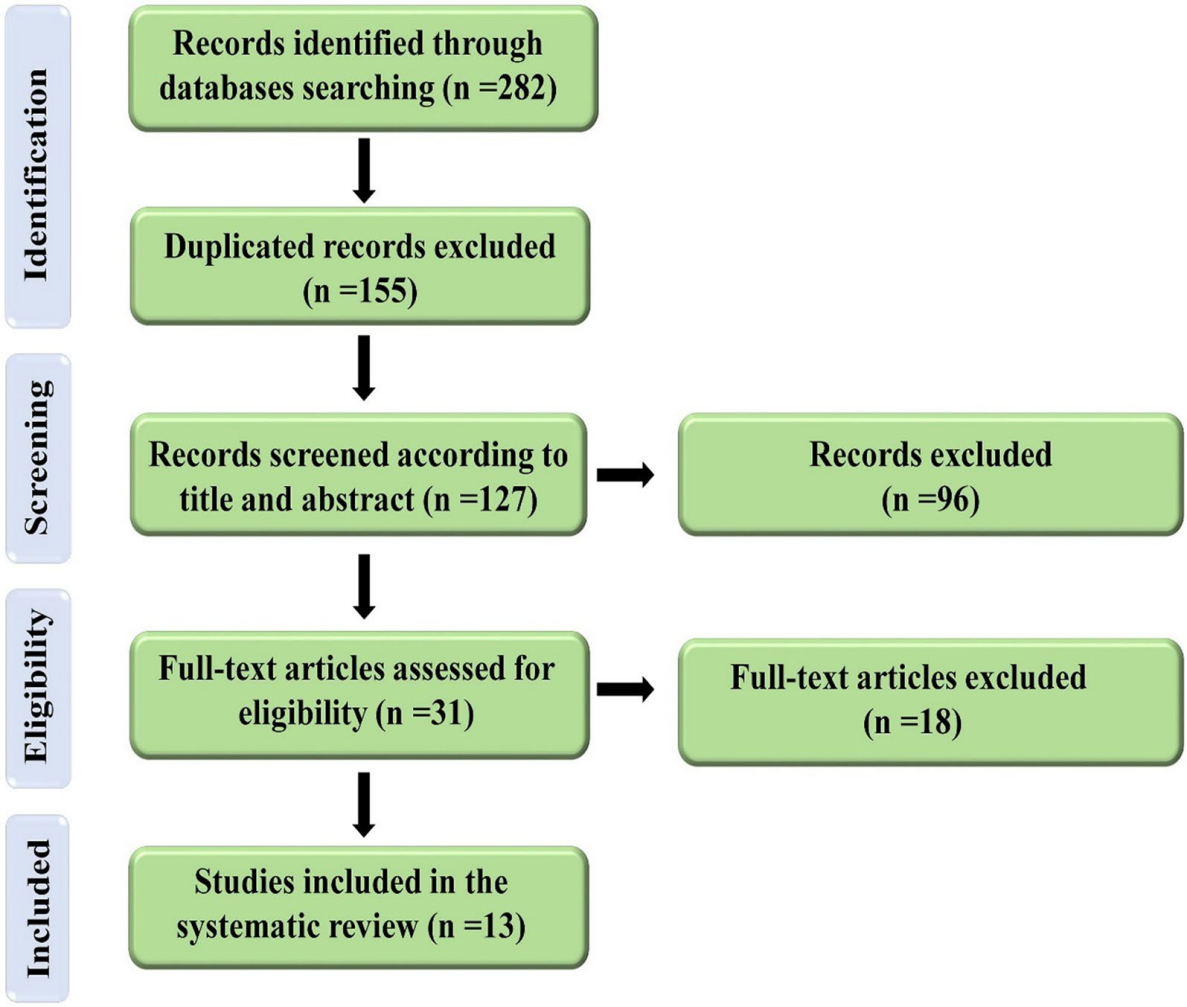
Flow diagram of PRISMA applied in the current systematic study for selection process

Figure 2 shows the process of study selection.

Two hundred and eighty-two articles were acquired by a comprehensive search on Web of Science (n = 112), Embase (n = 56), PubMed (n = 60), and Scopus (n = 54) electronic databases up to May 2021. After removing the duplicated papers (*n* = 155), the remaining ones (*n* = 127) were screened in their titles and abstracts, and 96 of them were omitted. Thirty-one articles were qualified for assessment of their full-texts. Thirteen articles were included in the current systematic review in accordance with the inclusion and exclusion criteria. The data of each eligible article were extracted and listed in Table 1.

**Table 1:** The characteristics of included studies

Author & year	Model	5-FU dosage & protocol of usage; route of administration	Outcomes of 5-FU chemotherapy drug	Resveratrol dosage & protocol of usage; route of administration	Resveratrol co-administration outcomes
Colin et al., 2009 [31]	In vitro/SW480 cells	1, 2, 5, and 10 μM & 24 h	↑cell proliferation inhibition	10 μM & co-treatment and 24 h prior to treatment	↑↑cell proliferation inhibition
Lee et al., 2010 [115]	In vitro/HCT116 cells	50 μM & 24 h	No change in caspase-6 level	200 μM & 24 h after treatment	↑caspase-6 activation
Mohapatra et al., 2011 [32]	In vitro/HCT116 cells	0.2, 0.5, 1.0, 2.0, 2.5, 5.0, and 10.0 μM & 24 h	↓cell survival, ↓cell migration, ↑apoptosis, ↑BAX, ↓Bcl-XL, ↑cleaved caspase-3, caspase-8, & caspase-9, ↑cleaved PARP, ↑cell population (%) in S phase, ↑DNA damage, ↑phospho-JNK & phospho-p38 levels	5, 10, 15, and 20 μM & 24 h prior to treatment	↓↓cell survival, ↓↓ migration, ↑↑apoptosis, ↑↑BAX, ↓↓Bcl-XL, ↑↑cleaved caspase-3, caspase-8, & caspase-9, ↑↑cleaved PARP, ↑↑cell population (%) in S phase, ↑↑DNA damage, ↑↑phospho-JNK & phospho-p38 levels
Santandreu et al., 2011 [33]	In vitro/HT-29 & SW-620 cells	10 µM & 6 h	↓cell viability in HT-29 cells, ↑intracellular ROS & LPO, ↓catalase & GPx/SOD ratio, ↓phospho-AKT & phospho-STAT3	100 μM & co-treatment	↓↓cell viability in both HT-29 & SW-620 cells, ↑↑intracellular ROS & LPO, ↓↓catalase & GPx/SOD ratio, ↓↓phospho-AKT & phospho-STAT3
Hotnog et al., 2013 [40]	In vitro/LoVo cells	25 µM & 24 h and 72 h	↑apoptosis (for both 24 h & 72 h treatment), ↑cell population (%) in G_0_/G_1_ phase, ↓cell population (%) in S phase, ↑P53 & BAX expression, ↓Bcl-2 expression	25, 50 and 100 μM & 6 h prior to treatment	↑↑apoptosis (for 24 h treatment) & ↓apoptosis (for 48 h treatment), ↑↑cell population (%) in G_0_/G_1_ phase (for 25 μM resveratrol + 25 µM 5-FU) & ↓cell population (%) in G_0_/G_1_ phase (for 100 μM resveratrol + 25 µM 5-FU), ↓↓cell population (%) in S phase, ↑↑P53 & BAX expression, ↓↓Bcl-2 expression
Kumazaki et al., 2013 [34]	In vitro/DLD-1, SW480 & COLO201 cells	1 µM & NI	↓cell viability, ↓phospho-Erk1/2, Erk1/2 & Sirt1 levels	10 µM & NI	↓↓cell viability, ↓↓ phospho-Erk1/2, Erk1/2 & Sirt1 levels
Buhrmann et al., 2015 [35]	In vitro/HCT116, SW480, HCT116^R^ & W480^R^ cells	0.01, 0.1, and 1 nM & 1–22 days	↓proliferation of HCT116 cells, ↓migration rate of HCT116 cells, ↓number of intercellular junctions in both HCT116 & HCT116^R^ cells, ↑apoptosis in HCT116 cells, ↓E-cadherin & claudin-2 proteins in both HCT116 & HCT116^R^ cells, ↑vimentin & transcription factor Slug expression in both HCT116 & HCT116^R^ cells, ↑NF-κB & IκBα phosphorylation in both HCT116 & HCT116^R^ cells, ↑activation of IKK, ↑MMP-9 & ↓cleaved caspase-3 in both HCT116 & HCT116^R^ cells	5 μM & co-treatment	↓↓proliferation of HCT116 & HCT116^R^ cells, ↓↓migration rate of HCT116 & HCT116^R^ cells, ↑number of intercellular junctions in both HCT116 & HCT116^R^ cells, ↑↑apoptosis in both HCT116 & HCT116^R^ cells, ↑E-cadherin & claudin-2 proteins in both HCT116 & HCT116^R^ cells, ↓vimentin & transcription factor Slug expression in both HCT116 & HCT116^R^ cells, ↓NF-κB & IκBα phosphorylation in both HCT116 & HCT116^R^ cells, ↓activation of IKK, ↓MMP-9 & ↑cleaved caspase-3 in both HCT116 & HCT116^R^ cells
Blanquer-Rossello et al., 2017 [36]	In vitro/SW620 cells	10 µM & NI	↑ROS, ↓cell viability	10 µM & NI	↑↑ROS, ↓↓cell viability
Buhrmann et al., 2018 [41]	In vitro/HCT116 & HCT116^R^ cells	0.1 and 1 nM & 10 days	↓invasion ability of HCT116 cells, ↓CD133, CD44 & ALDH1 in HCT116 cells, ↑apoptosis in HCT116 cells, ↑NF-κB activation, ↑MMP-9 & CXCR4 levels in HCT116 cells	5 µM; co-treatment	↓↓invasion ability of HCT116 & HCT116^R^ cells, ↓↓CD133, CD44 & ALDH1 in both HCT116 & HCT116^R^ cells, ↑↑apoptosis in both HCT116 & HCT116^R^ cells, ↓NF-κB activation, MMP-9 & CXCR4 levels in both HCT116 & HCT116^R^ cells, ↑cleaved caspase‐3 in both HCT116 & HCT116^R^ cells, ↑E-cadherin, ↓vimentin & transcription factor slug in both HCT116 & HCT116^R^ cells
Chung et al., 2018 [37]	In vitro/DLD1 & HCT116 cells	10 μM & 24 h and 72 h	↓cell proliferation, ↓population of HCT 116 cells (%) in G_0_/G_1_ phase, ↑population of HCT 116 cells (%) in S phase, ↑apoptosis in HCT 116 cells, ↓apoptosis in DLD1 cells, ↓transcription factor slug in both DLD1 & HCT116 cells, ↓CD51 in both DLD1 & HCT116 cells, ↓CD44 in HCT116 cells, ↓phospho-STAT3 & phospho-AKT in both DLD1 & HCT116 cells, ↓STAT3 binding to hTERT promoter region & telomerase activity in both DLD1 & HCT116 cells	25 μM & co-treatment	↑cytotoxicity, ↑population of HCT 116 cells in G_0_/G_1_ phase, ↓population of HCT 116 cells in S phase, ↓population of DLD1 cells in G_0_/G_1_ phase, ↑population of DLD1 cells in S phase, ↑↑apoptosis in both DLD1 & HCT116 cells, ↓↓ vimentin & transcription factor slug in HCT116 cells, ↓migration capacity (↑gap size) in both DLD1 & HCT116 cells, ↓↓ CD51 & CD44 in both DLD1 & HCT116 cells, ↓↓phospho-STAT3 & phospho-AKT in both DLD1 & HCT116 cells, ↓↓STAT3 binding to hTERT promoter region & telomerase activity in both DLD1 & HCT116 cells
Latif et al., 2019 [7]	In vivo/rats	12.5 mg/kg/day & on days 1, 3, and 5 with the cycle being repeated every 4 weeks over the duration of 4 months; *i.p*	↑MDA & AOPP, ↓SOD, ↑plasma NF-κB, ↓P53 expression, ↓COX-1 & COX-2 expression	10 mg/kg/day & co-treatment; oral	↓MDA & AOPP, ↑SOD, ↓plasma NF-κB, ↑P53 expression, ↓↓COX-1 & COX-2 expression
Hu et al., 2019 [39]	In vivo/mice	30 mg/kg/2 days & for 30 days; *i.p*	↓body weight, tumor volume & tumor weight, ↑inhibition rate, ↓vascular density, ↓CD31 & phospho-ERK, ↑COX-2 expression, ↑IL-6 & TNF-α levels	1920 mg/kg/day & co-treatment; *i.g*	↓↓body weight, tumor volume & tumor weight, ↑↑inhibition rate, ↓↓vascular density, ↓↓CD31 & phospho-ERK, ↓COX-2 expression, ↓IL-6 & TNF-α levels
Huang et al., 2019 [38]	In vitro/SW480, SW480 tumorigenic stem cells (SW480 CD133^+^), LoVo & LoVo tumorigenic stem cells (LoVo CD133^+^)	10, 20, 40, 80, 160, and 320 µM & 24 h	↓cell survival rate, ↓caspase‐3, ↑P53 & BAX expression	80, 160, &320 µM & co-treatment	↓↓cell survival rate, ↑caspase‐3, ↑↑P53 & BAX expression

↑, Increase; ↓, Decease; NI, Not informed; i.p., Intraperitoneal; i.g., Intragastrical; MDA, Malondialdehyde; ROS, Reactive oxygen; GPx, Glutathione peroxidase; SOD, Superoxide dismutase; JNK, c-Jun N-terminal kinase; COX, Cyclooxygenase; LPO, Lipid peroxidation; MMP-9, matrix metalloproteinase-9; PARP, Poly (ADP-ribose) polymerase; IKK, IκB kinase; AOPP, advanced oxidation protein products; BAX, Bcl-2-associated X protein; Bcl-xL, B-cell lymphoma-extra large; STAT3, Signal transducer and activator of transcription 3; ERK1/2, Extracellular signal-regulated kinase 1/2; NF-κB, Nuclear factor kappa B; ALDH1, Aldehyde Dehydrogenase 1; IL-6, Interleukin 6; TNF-α, Tumor necrosis factor alpha

### The effects of resveratrol in combination with 5-fluorouracil against colorectal cancer

#### Cell proliferation inhibition

The results obtained from some studies showed that proliferation inhibition of colorectal cancer cells in the groups treated by 5-FU was remarkably higher than the untreated groups [31–38]. Additionally, the inhibited cell proliferation following 5-FU treatment had time- and dose-dependent manners. In detail, it was observed that the colorectal cancer cell viability increases over time; while, the colorectal cancer cell viability decreases by increasing the chemotherapy dosage [31, 33, 35, 37, 38]. Furthermore, the type of colorectal cancer cell had a considerable effect on 5-FU-induced cell growth inhibition. For instance, it was shown that the SW-620 cells were more resistant to 5-FU than the HT-29 cells [33]. In addition, it was reported that the parental colorectal cancer cells (such as HCT116 and SW480) were more sensitive to 5-FU treatment than their corresponding isogenic FU-chemoresistant derived clones (i.e., HCT116R and SW480R) [35]. The resveratrol co-administration significantly increased cytotoxicity induced by 5-FU treatment on the colorectal cancer cells (synergistic effect) [31–38].

#### Tumor volume and tumor weight changes

The in vivo results demonstrated that tumor volume and tumor weight of mice were decreased in the 5-FU group than the untreated group. When both resveratrol and 5-FU were administered to the mice, dramatic decreases in the tumor volume and tumor weight were found compared to the 5-FU-treated group [39].

#### Cell cycle distribution

The findings revealed that the use of 5-FU chemotherapeutic agent regulated the cell cycle profile of colorectal cancer cells [32, 37, 40]. Chung et al. showed that upon 5-FU treatment, the HCT116 cells (%) in G_0_/G_1_ phase decreased, while the rate of these cells in S phase increased compared to the control group [37]. The combined treatment of resveratrol and 5-FU had a reverse manner on the cell cycle distribution compared with the chemotherapy group alone [37]. However, Mohapatra et al. showed that treatment with 5-FU alone and combined with resveratrol halted the cell cycle progression at the S phase in the HCT-116 cells [32].

It is noteworthy that the effect of combined treatment of 5-FU and resveratrol (compared with 5-FU alone) on the cell cycle distribution depends on the type of colorectal cancer cell and can provide different results.

#### Changes induced in biochemical markers

The biochemical changes induced by 5-FU treatment on colorectal cancer cells/tissues are listed in Table 1. It was found that intracellular reactive oxygen species (ROS), lipid peroxidation (LPO), malondialdehyde (MDA), superoxide dismutase (SOD), Bcl-2-associated X protein (BAX), CXCR4, MMP-9, cleaved poly (ADP-ribose) polymerase (PARP), phospho-JNK, phospho-p38, vimentin, activated nuclear factor kappa B, IκBα, IKK, interleukin 6 (IL-6), and tumor necrosis factor alpha (TNF-α) levels increased significantly in the 5-FU-treated groups than the untreated groups [7, 32, 33, 35, 36, 38–41]. In contrast, catalase, glutathione peroxidase (GPx)/SOD ratio, B-cell lymphoma-extra large (Bcl-xL), Bcl-2, phospho-AKT, phospho-signal transducer and activator of transcription 3 (STAT3), phospho-extracellular signal-regulated kinase (ERK)1/2, Sirt1, E-cadherin, claudin-2, aldehyde dehydrogenase isoform 1 (ALDH1), CD31, CD44, CD51, and CD133 levels decreased significantly [7, 32–35, 37, 39, 41].

The combined treatment of resveratrol and 5-FU synergized biochemical changes induced by 5-FU treatment alone (for most of the cases) [32–34, 36–41]. Nevertheless, it was observed that combination treatment of resveratrol and 5-FU compared to chemotherapy alone had a reverse manner on several biochemical markers (for instance, MDA, SOD, CXCR4, MMP-9, activated NF-κB, IκBα, IKK, vimentin, E-cadherin, claudin-2, IL-6, and TNF-α) [7, 35, 39, 41].

It is noteworthy that several studies have shown conflicting results on several biochemical markers (see Table 1). For instance, several studies showed decreased levels of cleaved caspase-3, P53 gene, transcription factor slug, and COX‐2 following 5-FU treatment alone [7, 35, 37, 38], while other studies revealed increased levels for these biomarkers [32, 35, 38–40]. Nevertheless, the combined treatment of resveratrol and 5-FU increased the cleaved caspase-3 and P53 gene levels and decreased the transcription factor slug and COX-2 levels, compared with chemotherapy groups alone [7, 32, 35, 37–41].

#### Histological changes

The histopathological results from the colon tissues of rats which received methylnitrosourea (as a tumor inducer) demonstrated moderate to severe inflammation, epithelial hyperplasia determined by crypt injury, and inflammatory cell infiltration. Nevertheless, the colon tissue sections from methylnitrosourea-induced colon cancer rats treated with 5-FU alone and 5-FU combined with resveratrol had more intact surface epithelium, less inflammatory, and normal colon cells than the methylnitrosourea-induced colon cancer group [7]. In another study, the histopathological findings obtained from mice with untreated colon tumors revealed blood vessels with a high vascular density; nevertheless, the vascular network of 5-FU-treated group illustrated significantly lower vascular density and branches and this decreased vascular density was severe in the group treated with resveratrol and 5-FU [39].

## Discussion

**Fig. 3:**
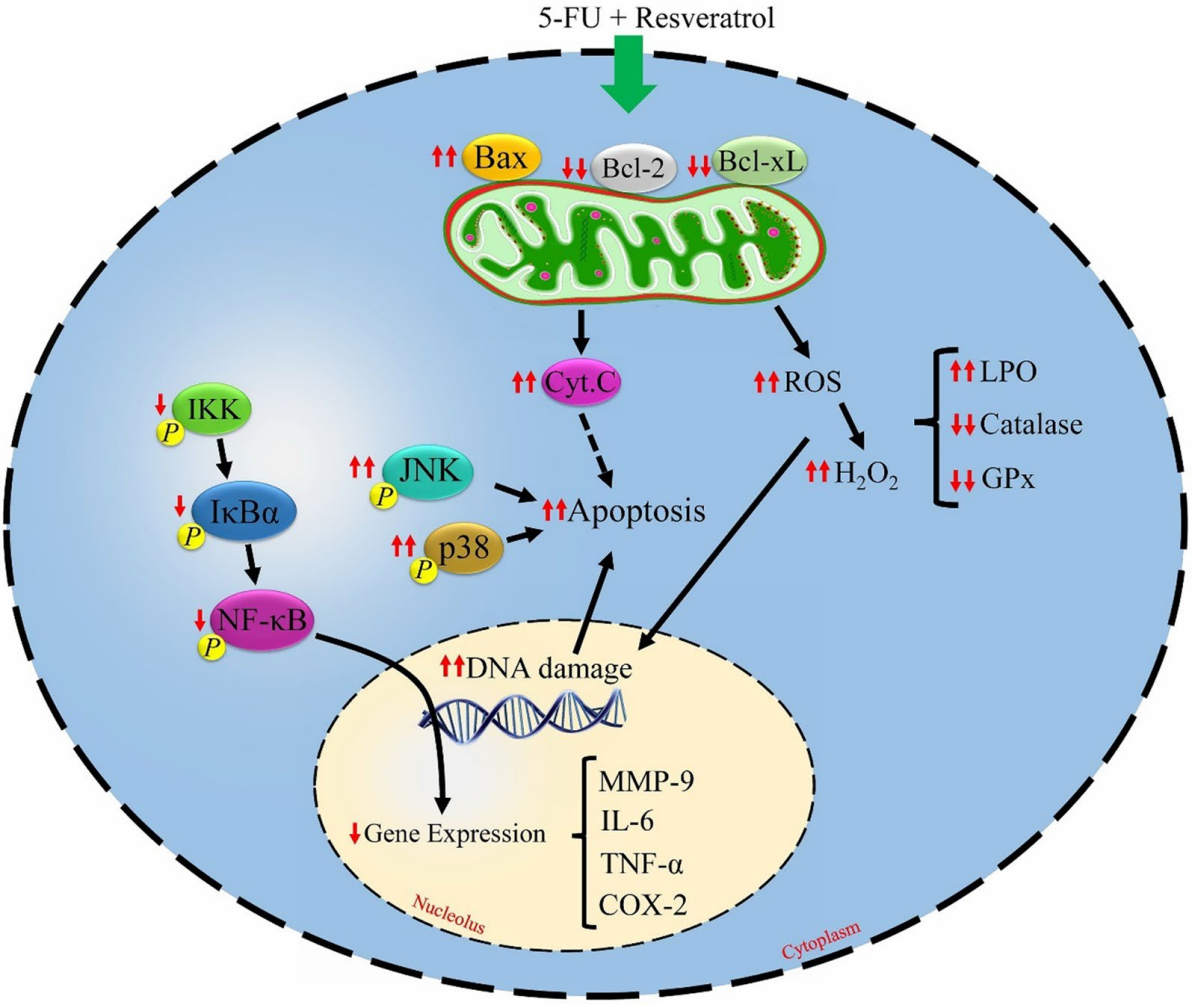
The molecular mechanisms of resveratrol co-treatment during colorectal cancer treatment by 5-fluorouracil (5-FU) chemotherapeutic agent. Resveratrol exerts the synergistic anti-tumoral effects through increments in oxidant and apoptosis activities, and reduction in inflammatory effects. ↑↑synergistically increased by 5-FU plus resveratrol compared to 5-FU alone; ↑↑synergistically decreased by 5-FU plus resveratrol compared to 5-FU alone; ↓decreased by 5-FU plus resveratrol compared to 5-FU alone; Bcl-xL, B-cell lymphoma-extra large; BAX, Bcl-2-associated X protein; GPx, glutathione peroxidase; IL-6, interleukin 6; IKK, IκBα kinase; LPO, lipid peroxidation; ROS, reactive oxygen species; NF-κB, nuclear factor-kappa B; COX-2, cyclooxygenase-2; TNF-α, tumor necrosis factor alpha.; MMP-9, matrix metalloproteinase-9

In the current systematic review, we aimed to investigate the cytotoxic effects induced by 5-FU treatment on colorectal cancer cells/tissues. Additionally, the potential therapeutic effects of resveratrol in combined with 5-FU were assessed. A summary of the results has been presented in Table 1. Moreover, the effects of resveratrol co-treatment during colorectal cancer treatment by 5-FU chemotherapeutic agent are depicted in Fig. 3.

Chemotherapy drugs is widely applied to treat different malignant tumors, such as head and neck, breast, gastric, and colorectal, etc. [42–45]. These drugs are able to modulate cell cycle that results in apoptosis [46–48]. The treatment of colorectal cancer with 5-FU (especially advanced cases) has two major problems: severe toxicity to normal tissues and development of tumor resistance [33]. As its dose increases, the unwanted adverse effects of the chemotherapeutic agent also increase and resistance to the agent develops frequently [8]. In addition, concomitant use of other chemotherapy drugs with 5-FU in colon cancer patients with advanced stages develops drug resistance against chemotherapy and these patients suffer from severe adverse effects [49, 50]. In view of the above, clinical application of 5-FU is limited during colorectal cancer treatment. These limitations highlight the requirement for the development of novel anti-tumoral agents in order to enhance the chemosensitivity of colorectal cancer cells and reduce unwanted adverse effects. In this regard, the combination treatment of 5-FU with less toxic substances derived from plants, such as resveratrol, has received more attention in recent years.

The anti-tumoral activity of resveratrol has been reported in some cancers, such as breast [51], skin [52], gastric [53], liver, and colorectal [54] cancers. More importantly, it has been shown that a combination of resveratrol and chemotherapeutic drugs not only can mitigate adverse drug reactions but can also reduce drug resistance (synergistic effect) [38, 55, 56]. Resveratrol exerts its anti-tumoral effects through several mechanisms, including oxidant, apoptotic, anti-inflammatory actions, etc. In the following, the mechanistic effects of 5-FU treatment on the colorectal cancer cells/tissues as well as the anti-tumoral effects of resveratrol co-treatment during colorectal cancer treatment by 5-FU chemotherapeutic agent are discussed.

### Oxidant actions

Free radicals are normally generated in the cells and several defense mechanisms protect the cells against them [57, 58]. In oxidative stress conditions, a lack of balance between the free radical amounts and these defense systems occurs, leading to increased free radical amounts [59, 60]. Following the chemotherapy drug administration, the oxidative stress condition happens [61]. The generated ROS following 5-FU treatment can induce DNA damage either directly or indirectly, leading to cancerous cell death [33, 36, 62]. It was also showed that LPO levels were increased in both the plasma and erythrocyte samples of 5-FU-treated colorectal cancer patients, whereas GPx and glutathione (GSH) levels were decreased [63]. Additionally, 5-FU significantly inhibited the catalase activity and increased SOD activity [33]. These findings reflect that 5-FU treatment impairs hydroperoxide scavenging capacity in colon cancer cells. It is noteworthy that non-radical ROS such as H_2_O_2_ through the activity of GPx enzyme and consuming GSH (as an important intracellular anti-oxidant agent) generates 2H_2_O [64]. Additionally, the catalase enzyme decomposes H_2_O_2_ to H_2_O and O_2_ [65]. O^2−^ also is one of the ROS molecules and it is turned to H_2_O_2_ by the SOD enzyme [66]. Moreover, the marked increases in advanced oxidation protein products and MDA levels of 5-FU-treated colon cancer rats were observed [7]. In contrast, the colorectal cancer cell lines resistant to 5-FU showed increments in glutathione transferases (GST) level and Nrf2 expression following 5-FU treatment [67, 68]. The activated Nrf2 also led to an increment in the protein expression and activity of heme oxygenase-1 (HO-1) [68]. Nrf2 is a transcription factor for several molecules involved in anti-oxidant activities, including NAD(P)H Quinone Dehydrogenase 1, HO-1, GST, and γ-glutamylcysteine synthetase (γ-GCS) [69]. Hence, an increased Nrf2 expression or Nrf2-regulated cytoprotective genes (such as GST) may play an important role in 5-FU resistance of cancerous cells.

Resveratrol through oxidant actions increased ROS level in colorectal cancer cells [33, 36]. It has been also shown that this herbal agent through the interaction with the mitochondria of malignant cells is able to induce an imbalance in cellular anti-oxidant activities, which results to a remarkable increment in the levels of both intracellular ROS and lipid peroxides [33]. It is noteworthy that free radicals are normally created in the cells, particularly through electrons leakage from the mitochondrial electron transport chain and this process is enhanced during mitochondria damage [70]. Resveratrol can also inhibit oxidation–reduction (redox) system in malignant cells [33]. Moreover, it can downregulate catalase and GPx expressions and upregulate MDA, SOD, and LPO levels in colorectal cancer cells [7, 33]. Resveratrol can also suppress Nrf2 activity, which may sensitize cancer cells resistance to chemotherapy agents in this way [71]. Besides, it was found that resveratrol combined with 5-FU chemotherapeutic drug against colorectal cancer synergistically increased the generated ROS, LPO, and SOD levels and decreased the catalase and GPx levels [33, 36].

### Apoptotic actions

Apoptosis is a physiological pathway which is needed to eliminate the harmed or transformed cells [72, 73]. It can happen during oxidative stress conditions and enormous DNA damage [74, 75]. Among the important medicators involved in the apoptosis process are caspase enzymes, Bcl-2, p53, BAX, Bcl-XL, ceramide, PARP, nuclear factor of activated T cells 5 (NFAT5), and so on [76–87]. One of the characteristics of most cancerous cells is the evasion of apoptosis; as any reduction or irregularity of this pathway invariably accompanies tumorigenesis and maintains malignant progression [88]. Some chemotherapeutic agents are able to induce apoptosis in tumoral cells [89, 90]. Several studies have reported an increased apoptosis level in 5-FU-treated colorectal cancer cells than untreated groups [32, 35, 41]. When colorectal cancer cells were treated to 5-FU, Bcl-XL expression was decreased compared to the untreated group [32]. In contrast, it was found that BAX, cleaved caspase-3, caspase-8, caspase-9, and PARP product levels were increased following 5-FU treatment [32, 38]. p53, tumor suppressor protein, has pro-apoptotic activities and 50–70% of colorectal tumors contain p53 mutation [91]. It was shown an increased level of P53 expression following 5-FU treatment [92]. These indicate that colorectal cancer cells are moving towards apoptosis. PARP is a nuclear enzyme and, as a pro-apoptotic factor, can be activated by chemotherapeutic agents [32, 93–95]. It has been also reported that 5-FU damages DNA and takes the colon cancer cells to apoptosis by activating the MAPK pathway [96]. In this regard, it was found that 5-FU activates (phosphorylates) phospho-JNK and phospho-p38 (major MAPK signaling components) [32]. MAPKs may also affect NFAT5 [97–100] and p53 [101] activities. NFAT5 is a transcription factor which plays a critical role in apoptosis [86]. Moreover, it was shown that 5-FU induces apoptosis through suppression of the MAPK/ERK1/2 signaling pathway in colon cancerous cells [34].

Some studies have reported that resveratrol can induce apoptosis in various malignant tumor cells, such as glioma [102], prostate [103], gastric [104], breast [105], head and neck [106], and ovarian [107] cancer cells. The apoptotic activation of resveratrol is linked to induce ROS production, various caspases activation, mitochondrial membrane permeability, BAX and p53 activation, etc. [108–113]. It has been reported that colorectal cancer cells treated with resveratrol revealed an increased apoptosis level compared to untreated groups [32, 35, 114, 115]. The use of resveratrol decreased Bcl-XL expression in the cancerous cells, while it led to increased levels of BAX, cleaved caspase-3, caspase-6, caspase-8, caspase-9, and PARP product [32, 35, 38]. It was also result to increase p53, phospho-JNK, and phospho-p38 levels in colorectal cancer cells [7, 32, 38]. Additionally, resveratrol suppressed the MAPK/ERK1/2 signaling pathway in colon cancerous cells, leading to increase apoptosis [34]. Moreover, the results represented in this study showed that the use of resveratrol could synergize 5-FU-induced apoptosis in colorectal cancer cell lines [32, 35, 37].

### Inflammatory actions

The inflammatory process is a biological phenomenon which occurs in response to tissue damage induced from several harmful stimuli such as chemotherapy, radiation, microbial pathogen infection, and/or wounding [116–121]. Among the characteristics of inflammation are: elevation in leukocyte migration to injured area, upregulation of pro-inflammatory cytokines, and leukocyte chemotaxis [122, 123]. The chemotherapy-induced inflammation has a main role in tumor resistance and the incidence of various adverse effects. Additionally, chronic inflammation may develop second cancer during years after treatment [124]. Moreover, inflammatory mediators play an important role in angiogenesis and tumor growth. For instance, NF‐κB is considered as one of the most significant links between inflammation, tumor resistance, and cell death [124]. It was found chemotherapy by 5-FU increases NF‐κB phosphorylation in colorectal cancer cells [7, 35, 41, 124]. In addition, 5-FU markedly induced phosphorylation of IκBα subunit, expression of MMP-9, and activation of IKK [35, 41, 125]. The MMP-9, as an enzyme involved in the inflammatory pathway, is regulated by NF-κB [125]. IkB inhibits nuclear translocation of NF-κB and also activation of IKK induces phosphorylation of NF-κB and IκB [85]. NF‐κB can also stimulate the release of prostaglandins and resistance of cancerous cells to apoptosis through upregulation of COX‐2 [124]. The findings obtained from 5-FU-treated mice also showed an increased expression of COX-2 than the control group [39]. COX‐2 is a pro-inflammatory enzyme that is overexpressed at the inflammatory site of cancer [126]. It is a marker representing a worse prognosis and a stimulator for various cancers through multitasking roles (recently discussed in detail by Hashemi Goradel et al. [127]). It is noteworthy that suppression of NF‐κB and COX‐2 by novel therapeutic agents could provide a promising approach for cancer treatment, particularly when they are applied as an adjuvant with appropriate chemotherapy drugs. It has been also reported that the phosphorylated STAT3 level decreases in response to 5-FU treatment than the control levels of colorectal cancer cells [33, 37]. One of the reasons for the resistance of cancerous cells is the upregulation of transcription factors such as STATs [16]. These enzymes are considered as transcription factors for cytokine signaling which are constitutively activated in many cancer types [128–131]. STAT3, as one of the subfamilies, involves in the modulation of angiogenesis and metastasis, suppression of apoptosis, and regulation of cell cycle progression via stimulation of VEGF, MMP-2, MMP-9, and IAP-1 [16, 132]. Of note, targeting the STATs by chemotherapy agents can be considered as a strategy for overcoming tumor resistance. Moreover, the increased levels of IL-6 and TNF-α following 5-FU-treated colorectal cancer cells have been reported [39].

As mentioned earlier, inflammation is an inducer of carcinogenesis and can significantly affect the therapeutic outcome of chemotherapy [127, 133]. In this regard, resveratrol, through its anti-inflammatory activities, can increase therapeutic efficiency and reduce the resistance of cancer cells to chemotherapy drugs. According to the findings obtained from combined treatment of resveratrol and 5-FU, it was found that resveratrol reduces phosphorylated NF‐κB, IκBα, and IKK levels as well as MMP-9, COX-2, IL-6, and TNF-α levels in colorectal cancer cells [7, 35, 39, 41]. Resveratrol also synergistically reduced phosphorylated STAT3 levels in 5-FU-treated colorectal cancer cells [33, 37].

## Perspective of future research

In addition to its anti-tumoral effects, resveratrol is able to mitigate chemotherapy-induced toxicities in normal cells/tissues. Resveratrol exerts the chemo-protective effects through anti-oxidant, anti-apoptotic, anti-inflammatory actions. Hence, it is proposed to conduct studies on the chemo-protective effects of resveratrol in normal cells/tissues during colorectal cancer chemotherapy by 5-FU.

It is noteworthy that the results represented in this systematic review are in accordance with in vitro and in vivo models. The use of resveratrol as a chemo-protector agent or/and chemo-sensitizer agent combined to 5-FU in colorectal cancer patients needs further studies because sometimes results are different between the in vitro and in vivo models and clinical studies.

## Conclusion

The obtained findings revealed that combined treatment of resveratrol and 5-FU chemotherapeutic drug significantly increases the effectiveness of 5-FU treatment alone in colorectal cancer cells through increment of chemosensitizer and/or reduction of chemoresistance effects. Resveratrol, as an anti-tumoral agent, exerts the synergistic effects through various mechanisms such as regulation of cell cycle distribution, increments in oxidant and apoptosis activities, and reduction in inflammatory effects.

## Data Availability

The datasets used and/or analyzed during the current study are available from the corresponding author on reasonable request.

## References

[1] Mohammadianpanah MJ (2015). Colorectal cancer incidence: does Iran follow the West?. Iran J Colorect Res.

[2] Bray F, Ferlay J, Soerjomataram I, Siegel RL, Torre LA, Jemal AJ (2018). Global cancer statistics 2018: GLOBOCAN estimates of incidence and mortality worldwide For 36 cancers in 185 countries. CA..

[3] Farhood B, Raei B, Malekzadeh R, Shirvani M, Najafi M, Mortezazadeh T (2019). A review of incidence and mortality of colorectal, lung, liver, thyroid, and bladder cancers in Iran and compared to other countries. Contemp Oncol.

[4] Mármol I, Sánchez-de-Diego C, Pradilla Dieste A, Cerrada E, Rodriguez Yoldi MJ (2017). Colorectal carcinoma: a general overview and future perspectives in colorectal cancer. Int J Mol Sci.

[5] Goldstein DA, Zeichner SB, Bartnik CM, Neustadter E, Flowers CR (2016). Metastatic colorectal cancer: a systematic review of the value of current therapies. Clin Color Cancer..

[6] Deng Z, Qin Y, Wang J, Wang G, Lang X, Jiang J, Xie K, Zhang W, Xu H, Shu Y, Zhang Y (2020). Prognostic and predictive role of DNA mismatch repair status in stage II-III colorectal cancer: a systematic review and meta-analysis. Clin Genet..

[7] Abdel Latif Y, El-Bana M, Hussein J, El-Khayat Z, Farrag AR (2019). Effects of resveratrol in combination with 5-fluorouracil on N-methylnitrosourea-induced colon cancer in rats. Comp Clin Pathol.

[8] Katona C, Kralovánszky J, Rosta A, Pandi E, Fónyad G, Tóth K, Jeney A (1998). Putative role of dihydropyrimidine dehydrogenase in the toxic side effect of 5-fluorouracil in colorectal cancer patients. Oncology.

[9] Salehi B, Mishra AP, Nigam M, Sener B, Kilic M, Sharifi-Rad M, Fokou PVT, Martins N, Sharifi-Rad J (2018). Resveratrol: a double-edged sword in health benefits. Biomedicines.

[10] Yahyapour R, Shabeeb D, Cheki M, Musa AE, Farhood B, Rezaeyan A, Amini P, Fallah H, Najafi M (2018). Radiation protection and mitigation by natural antioxidants and flavonoids: implications to radiotherapy and radiation disasters. Curr Mol Pharmacol.

[11] Gusman J, Malonne H, Atassi G (2001). A reappraisal of the potential chemopreventive and chemotherapeutic properties of resveratrol. Carcinogenesis.

[12] Pervaiz S, Holme AL (2009). Resveratrol: its biologic targets and functional activity. Antioxid Redox Signal.

[13] Thomas E, Gopalakrishnan V, Hegde M, Kumar S, Karki SS, Raghavan SC, Choudhary B (2016). A novel resveratrol based tubulin inhibitor induces mitotic arrest and activates apoptosis in cancer cells. Sci Rep.

[14] Ahmadi Z, Mohammadinejad R, Ashrafizadeh M (2019). Drug delivery systems for resveratrol, a non-flavonoid polyphenol: Emerging evidence in last decades. J Drug Del Sci Technol.

[15] de la Lastra CA, Villegas I (2007). Resveratrol as an antioxidant and pro-oxidant agent: mechanisms and clinical implications. Biochem Soc Trans.

[16] Mortezaee K, Najafi M, Farhood B, Ahmadi A, Shabeeb D, Musa AE (2020). Resveratrol as an adjuvant for normal tissues protection and tumor sensitization. Curr Cancer Drug Targets.

[17] Kisková T, Kassayová M (2019). Resveratrol action on lipid metabolism in cancer. Int J Mol Sci.

[18] Honari M, Shafabakhsh R, Reiter RJ, Mirzaei H, Asemi Z (2019). Resveratrol is a promising agent for colorectal cancer prevention and treatment: focus on molecular mechanisms. Cancer Cell Int.

[19] Ko JH, Sethi G, Um JY, Shanmugam MK, Arfuso F, Kumar AP, Bishayee A, Ahn KS (2017). The role of resveratrol in cancer therapy. Int J Mol Sci.

[20] Xiao Q, Zhu W, Feng W, Lee SS, Leung AW, Shen J, Gao L, Xu C (2018). A review of resveratrol as a potent chemoprotective and synergistic agent in cancer chemotherapy. Front Pharmacol.

[21] Soliman BA, Farrag ARH, Khaled HAS, Mohamed AAM (2018). Combinational effect of 5-flourouracil and resveratrol against N-nitroso-N-methyl urea induced colorectal cancer. Egypt J Hosp Med.

[22] Varoni EM, Lo Faro AF, Sharifi-Rad J, Iriti M (2016). Anticancer molecular mechanisms of resveratrol. Front Nutr.

[23] van Ginkel PR, Sareen D, Subramanian L, Walker Q, Darjatmoko SR, Lindstrom MJ, Kulkarni A, Albert DM, Polans AS (2007). Resveratrol inhibits tumor growth of human neuroblastoma and mediates apoptosis by directly targeting mitochondria. Clin Cancer Res.

[24] Hu LF, Lan HR, Li XM, Jin KT (2021). A Systematic review of the potential chemoprotective effects of resveratrol on doxorubicin-induced cardiotoxicity: focus on the antioxidant, antiapoptotic, and anti-inflammatory activities. Oxid Med Cell Longev.

[25] Kundu JK, Surh YJ (2008). Cancer chemopreventive and therapeutic potential of resveratrol: mechanistic perspectives. Cancer Lett.

[26] Li D, Wang G, Jin G, Yao K, Zhao Z, Bie L, Guo Y, Li N, Deng W, Chen X (2019). Resveratrol suppresses colon cancer growth by targeting the AKT/STAT3 signaling pathway. Int J Mol Med.

[27] Ji Q, Liu X, Fu X, Zhang L, Sui H, Zhou L, Sun J, Cai J, Qin J, Ren J (2013). Resveratrol inhibits invasion and metastasis of colorectal cancer cells via MALAT1 mediated Wnt/β-catenin signal pathway. PLoS ONE.

[28] Whitlock NC, Baek SJ (2012). The anticancer effects of resveratrol: modulation of transcription factors. Nutr Cancer.

[29] Athar M, Back JH, Kopelovich L, Bickers DR, Kim AL (2009). Multiple molecular targets of resveratrol: anti-carcinogenic mechanisms. Arch Biochem Biophys.

[30] Moher D, Liberati A, Tetzlaff J, Altman DG (2009). Preferred reporting items for systematic reviews and meta-analyses: the PRISMA statement. Ann Intern Med.

[31] Colin D, Gimazane A, Lizard G, Izard JC, Solary E, Latruffe N, Delmas D (2009). Effects of resveratrol analogs on cell cycle progression, cell cycle associated proteins and 5fluoro-uracil sensitivity in human derived colon cancer cells. Int J Cancer.

[32] Mohapatra P, Preet R, Choudhuri M, Choudhuri T, Kundu CN (2011). 5-fluorouracil increases the chemopreventive potentials of resveratrol through DNA damage and MAPK signaling pathway in human colorectal cancer cells. Oncol Res.

[33] Santandreu FM, Valle A, Oliver J, Roca P (2011). Resveratrol potentiates the cytotoxic oxidative stress induced by chemotherapy in human colon cancer cells. Cell Physiol Biochem.

[34] Kumazaki M, Noguchi S, Yasui Y, Iwasaki J, Shinohara H, Yamada N, Akao Y (2013). Anti-cancer effects of naturally occurring compounds through modulation of signal transduction and miRNA expression in human colon cancer cells. J Nutr Biochem.

[35] Buhrmann C, Shayan P, Kraehe P, Popper B, Goel A, Shakibaei M (2015). Resveratrol induces chemosensitization to 5-fluorouracil through up-regulation of intercellular junctions, Epithelial-to-mesenchymal transition and apoptosis in colorectal cancer. Biochem Pharmacol.

[36] Blanquer-Rosselló MD, Hernández-López R, Roca P, Oliver J, Valle A (2017). Resveratrol induces mitochondrial respiration and apoptosis in SW620 colon cancer cells. Biochim Biophys Acta.

[37] Chung SS, Dutta P, Austin D, Wang P, Awad A, Vadgama JV (2018). Combination of resveratrol and 5-flurouracil enhanced anti-telomerase activity and apoptosis by inhibiting STAT3 and Akt signaling pathways in human colorectal cancer cells. Oncotarget.

[38] Huang L, Zhang S, Zhou J, Li X (2019). Effect of resveratrol on drug resistance in colon cancer chemotherapy. RSC Adv.

[39] Hu WH, Chan GK, Duan R, Wang HY, Kong XP, Dong TT, Tsim KW (2019). Synergy of ginkgetin and resveratrol in suppressing vegf-induced angiogenesis: a therapy in treating colorectal cancer. Cancers.

[40] Hotnog D, Mihăilă M, Lancu IV, Matei GG, Hotnog C, Anton G, Bostan M, Braşoveanu LI (2013). Resveratrol modulates apoptosis in 5-fluorouracyl treated colon cancer cell lines. Roum Arch Microbiol Immunol.

[41] Buhrmann C, Yazdi M, Popper B, Shayan P, Goel A, Aggarwal BB, Shakibaei M (2018). Resveratrol chemosensitizes TNF-β-induced survival of 5-FU-treated colorectal cancer cells. Nutrients.

[42] Zhang N, Yin Y, Xu SJ, Chen WS (2008). 5-Fluorouracil: mechanisms of resistance and reversal strategies. Molecules (Basel, Switzerland).

[43] Kumar BS, Ravi K, Verma AK, Fatima K, Hasanain M, Singh A, Sarkar J, Luqman S, Chanda D, Negi AS (2017). Synthesis of pharmacologically important naphthoquinones and anticancer activity of 2-benzyllawsone through DNA topoisomerase-II inhibition. Bioorg Med Chem.

[44] Sathish Kumar B, Singh A, Kumar A, Singh J, Hasanain M, Singh A, Masood N, Yadav DK, Konwar R, Mitra K (2014). Synthesis of neolignans as microtubule stabilisers. Bioorg Med Chem.

[45] Srivastava A, Fatima K, Fatima E, Singh A, Singh A, Shukla A, Luqman S, Shanker K, Chanda D, Khan F (2020). Fluorinated benzylidene indanone exhibits antiproliferative activity through modulation of microtubule dynamics and antiangiogenic activity. Eur J Pharm Sci.

[46] Liu HC, Chen GG, Vlantis AC, Leung BC, Tong MC, van Hasselt CA (2006). 5-fluorouracil mediates apoptosis and G1/S arrest in laryngeal squamous cell carcinoma via a p53-independent pathway. Cancer J.

[47] Khwaja S, Fatima K, Hasanain M, Behera C, Kour A, Singh A, Luqman S, Sarkar J, Chanda D, Shanker K (2018). Antiproliferative efficacy of curcumin mimics through microtubule destabilization. Eur J Med Chem.

[48] Sathish Kumar B, Kumar A, Singh J, Hasanain M, Singh A, Fatima K, Yadav DK, Shukla V, Luqman S, Khan F (2014). Synthesis of 2-alkoxy and 2-benzyloxy analogues of estradiol as anti-breast cancer agents through microtubule stabilization. Eur J Med Chem.

[49] Bastos DA, Ribeiro SC, de Freitas D, Hoff PM (2010). Combination therapy in high-risk stage II or stage III colon cancer: current practice and future prospects. Therap Adv Med Oncol.

[50] Panczyk M (2014). Pharmacogenetics research on chemotherapy resistance in colorectal cancer over the last 20 years. World J Gastroenterol.

[51] Chottanapund S, Van Duursen MB, Navasumrit P, Hunsonti P, Timtavorn S, Ruchirawat M, Van den Berg M (2014). Anti-aromatase effect of resveratrol and melatonin on hormonal positive breast cancer cells co-cultured with breast adipose fibroblasts. Toxicol In Vitro.

[52] Junco JJ, Mancha A, Malik G, Wei SJ, Kim DJ, Liang H, Slaga TJ (2013). Resveratrol and P-glycoprotein inhibitors enhance the anti-skin cancer effects of ursolic acid. Mol Cancer Res.

[53] Wang Z, Li W, Meng X, Jia B (2012). Resveratrol induces gastric cancer cell apoptosis via reactive oxygen species, but independent of sirtuin1. Clin Exp Pharmacol Physiol.

[54] Amiri F, Zarnani AH, Zand H, Koohdani F, Jeddi-Tehrani M, Vafa M (2013). Synergistic anti-proliferative effect of resveratrol and etoposide on human hepatocellular and colon cancer cell lines. Eur J Pharmacol.

[55] Chen YZ, Li ZD, Gao F, Zhang Y, Sun H, Li PP (2009). Effects of combined Chinese drugs and chemotherapy in treating advanced non-small cell lung cancer. Chin J Integr Med.

[56] Cocetta V, Quagliariello V, Fiorica F, Berretta M, Montopoli M (2021). Resveratrol as chemosensitizer agent: state of art and future perspectives. Int J Mol Sci.

[57] Nobakht-Haghighi N, Rahimifard M, Baeeri M, Rezvanfar MA, Moini Nodeh S, Haghi-Aminjan H, Hamurtekin E, Abdollahi M (2018). Regulation of aging and oxidative stress pathways in aged pancreatic islets using alpha-lipoic acid. Mol Cell Biochem.

[58] Haghi-Aminjan H, Asghari MH, Farhood B, Rahimifard M, Hashemi Goradel N, Abdollahi M (2018). The role of melatonin on chemotherapy-induced reproductive toxicity. J Pharm Pharmacol.

[59] Narayanaswamy PB, Hodjat M, Haller H, Dumler I, Kiyan Y (2014). Loss of urokinase receptor sensitizes cells to DNA damage and delays DNA repair. PLoS ONE.

[60] Momtaz S, Baeeri M, Rahimifard M, Haghi-Aminjan H, Hassani S, Abdollahi M (2019). Manipulation of molecular pathways and senescence hallmarks by natural compounds in fibroblast cells. J Cell Biochem.

[61] Gautam Y, Dwivedi S, Srivastava A, Singh A, Chanda D, Singh J, Rai S, Konwar R, Negi AS (2016). 2-(3′, 4′-Dimethoxybenzylidene) tetralone induces anti-breast cancer activity through microtubule stabilization and activation of reactive oxygen species. RSC Adv.

[62] Olivier C, Oliver L, Lalier L, Vallette FM (2020). Drug resistance in glioblastoma: the two faces of oxidative stress. Front Mol Biosci.

[63] Koçer M, Nazıroğlu M (2013). Effects of 5-fluorouracil on oxidative stress and calcium levels in the blood of patients with newly diagnosed colorectal cancer. Biol Trace Elem Res.

[64] Day BJ (2009). Catalase and glutathione peroxidase mimics. Biochem Pharmacol.

[65] Shrestha B, Reed JM, Starks PT, Kaufman GE, Goldstone JV, Roelke ME, O'Brien SJ, Koepfli KP, Frank LG, Court MH (2011). Evolution of a major drug metabolizing enzyme defect in the domestic cat and other felidae: phylogenetic timing and the role of hypercarnivory. PLoS ONE.

[66] Juarez JC, Manuia M, Burnett ME, Betancourt O, Boivin B, Shaw DE, Tonks NK, Mazar AP, Doñate F (2008). Superoxide dismutase 1 (SOD1) is essential for H2O2-mediated oxidation and inactivation of phosphatases in growth factor signaling. Proc Natl Acad Sci USA.

[67] Akhdar H, Loyer P, Rauch C, Corlu A, Guillouzo A, Morel F (2009). Involvement of Nrf2 activation in resistance to 5-fluorouracil in human colon cancer HT-29 cells. Eur J Cancer.

[68] Kang KA, Piao MJ, Kim KC, Kang HK, Chang WY, Park IC, Keum YS, Surh YJ, Hyun JW (2014). Epigenetic modification of Nrf2 in 5-fluorouracil-resistant colon cancer cells: involvement of TET-dependent DNA demethylation. Cell Death Dis.

[69] Kleszczyński K, Zillikens D, Fischer TW (2016). Melatonin enhances mitochondrial ATP synthesis, reduces reactive oxygen species formation, and mediates translocation of the nuclear erythroid 2-related factor 2 resulting in activation of phase-2 antioxidant enzymes (γ-GCS, HO-1, NQO1) in ultraviolet radiation-treated normal human epidermal keratinocytes (NHEK). J Pineal Res.

[70] Niaz K, Hassan FI, Mabqool F, Khan F, Momtaz S, Baeeri M, Navaei-Nigjeh M, Rahimifard M, Abdollahi M (2017). Effect of styrene exposure on plasma parameters, molecular mechanisms and gene expression in rat model islet cells. Environ Toxicol Pharmacol.

[71] Tian Y, Song W, Li D, Cai L, Zhao Y (2019). Resveratrol as a natural regulator of autophagy for prevention and treatment of cancer. Onco Targets Ther.

[72] Najafi M, Mortezaee K, Rahimifard M, Farhood B, Haghi-Aminjan H (2020). The role of curcumin/curcuminoids during gastric cancer chemotherapy: a systematic review of non-clinical study. Life Sci.

[73] Haghi-Aminjan H, Baeeri M, Rahimifard M, Alizadeh A, Hodjat M, Hassani S, Asghari MH, Abdollahi A, Didari T, Hosseini R (2018). The role of minocycline in alleviating aluminum phosphide-induced cardiac hemodynamic and renal toxicity. Environ Toxicol Pharmacol.

[74] Oben KZ, Gachuki BW, Alhakeem SS, McKenna MK, Liang Y, St Clair DK, Rangnekar VM, Bondada S (2017). Radiation Induced Apoptosis of Murine Bone Marrow Cells Is Independent of Early Growth Response 1 (EGR1). PLoS ONE.

[75] Komarova EA, Kondratov RV, Wang K, Christov K, Golovkina TV, Goldblum JR, Gudkov AV (2004). Dual effect of p53 on radiation sensitivity in vivo: p53 promotes hematopoietic injury, but protects from gastro-intestinal syndrome in mice. Oncogene.

[76] Mortezaee K, Najafi M, Farhood B, Ahmadi A, Potes Y, Shabeeb D, Musa AE (2019). Modulation of apoptosis by melatonin for improving cancer treatment efficiency: an updated review. Life Sci.

[77] Sogwagwa N, Davison G, Khan S, Solomon W (2016). P9 Correlation of radiation induced apoptosis with Bax and Bcl-2 protein expression. Physica Medica.

[78] Huerta S, Gao X, Dineen S, Kapur P, Saha D, Meyer J (2013). Role of p53, Bax, p21, and DNA-PKcs in radiation sensitivity of HCT-116 cells and xenografts. Surgery.

[79] Werner LR, Huang S, Francis DM, Armstrong EA, Ma F, Li C, Iyer G, Canon J, Harari PM (2015). Small molecule inhibition of MDM2-p53 interaction augments radiation response in human tumors. Mol Cancer Ther.

[80] Csuka O, Remenár E, Koronczay K, Doleschall Z, Németh G (1997). Predictive value of p53, Bcl2 and bax in the radiotherapy of head and neck cancer. Pathol Oncol Res.

[81] Maebayashi K, Mitsuhashi N, Takahashi T, Sakurai H, Niibe H (1999). p53 mutation decreased radiosensitivity in rat yolk sac tumor cell lines. Int J Radiat Oncol Biol Phys.

[82] Sugihara T, Murano H, Nakamura M, Ichinohe K, Tanaka K (2011). p53-Mediated gene activation in mice at high doses of chronic low-dose-rate γ radiation. Radiat Res.

[83] Punnoose EA, Leverson JD, Peale F, Boghaert ER, Belmont LD, Tan N, Young A, Mitten M, Ingalla E, Darbonne WC (2016). Expression Profile of BCL-2, BCL-XL, and MCL-1 predicts pharmacological response to the BCL-2 selective antagonist venetoclax in multiple myeloma models. Mol Cancer Ther.

[84] Haimovitz-Friedman A, Kolesnick RN, Fuks Z (1997). Ceramide signaling in apoptosis. Br Med Bull.

[85] Najafi M, Hooshangi Shayesteh MR, Mortezaee K, Farhood B, Haghi-Aminjan H (2020). The role of melatonin on doxorubicin-induced cardiotoxicity: a systematic review. Life Sci.

[86] Kim H, Yoo WS, Jung JH, Jeong BK, Woo SH, Kim JH, Kim SJ (2019). Alpha-Lipoic Acid Ameliorates Radiation-Induced Lacrimal Gland Injury through NFAT5-Dependent Signaling. Int J Mol Sci.

[87] Singh A, Fatima K, Singh A, Behl A, Mintoo MJ, Hasanain M, Ashraf R, Luqman S, Shanker K, Mondhe DM (2015). Anticancer activity and toxicity profiles of 2-benzylidene indanone lead molecule. Eur J Pharm Sci.

[88] Arabzadeh A, Mortezazadeh T, Aryafar T, Gharepapagh E, Majdaeen M, Farhood B (2021). Therapeutic potentials of resveratrol in combination with radiotherapy and chemotherapy during glioblastoma treatment: a mechanistic review. Cancer Cell Int.

[89] Krakstad C, Chekenya M (2010). Survival signalling and apoptosis resistance in glioblastomas: opportunities for targeted therapeutics. Mol Cancer.

[90] Hamid AA, Kaushal T, Ashraf R, Singh A, Chand Gupta A, Prakash O, Sarkar J, Chanda D, Bawankule DU, Khan F (2017). (22β,25R)-3β-Hydroxy-spirost-5-en-7-iminoxy-heptanoic acid exhibits anti-prostate cancer activity through caspase pathway. Steroids.

[91] Shen H, Maki CG (2011). Pharmacologic activation of p53 by small-molecule MDM2 antagonists. Curr Pharm Des.

[92] Huang L, Zhang S, Zhou J, Li X (2019). Effect of resveratrol on drug resistance in colon cancer chemotherapy. RSC Adv..

[93] Tentori L, Graziani G (2005). Chemopotentiation by PARP inhibitors in cancer therapy. Pharmacol Res.

[94] Azuma M, Yamashita T, Aota K, Tamatani T, Sato M (2001). 5-Fluorouracil suppression of NF-KappaB is mediated by the inhibition of IKappab kinase activity in human salivary gland cancer cells. Biochem Biophys Res Commun.

[95] Hamid AA, Hasanain M, Singh A, Bhukya B, Omprakash, Vasudev PG, Sarkar J, Chanda D, Khan F, Aiyelaagbe OO, et al. Synthesis of novel anticancer agents through opening of spiroacetal ring of diosgenin. Steroids 2014, 87:108–118. 10.1016/j.steroids.2014.05.025 24929045

[96] Bragado P, Armesilla A, Silva A, Porras A (2007). Apoptosis by cisplatin requires p53 mediated p38alpha MAPK activation through ROS generation. Apoptosis.

[97] Lee JH, Kim M, Im YS, Choi W, Byeon SH, Lee HK (2008). NFAT5 induction and its role in hyperosmolar stressed human limbal epithelial cells. Invest Ophthalmol Vis Sci.

[98] Lee N, Kim D, Kim WU (2019). Role of NFAT5 in the immune system and pathogenesis of autoimmune diseases. Front Immunol.

[99] Takeda K, Matsuzawa A, Nishitoh H, Ichijo H (2003). Roles of MAPKKK ASK1 in stress-induced cell death. Cell Struct Funct.

[100] Chang L, Karin M (2001). Mammalian MAP kinase signalling cascades. Nature.

[101] Brown L, Benchimol S (2006). The involvement of MAPK signaling pathways in determining the cellular response to p53 activation: cell cycle arrest or apoptosis. J Biol Chem.

[102] Lin HY, Tang HY, Keating T, Wu YH, Shih A, Hammond D, Sun M, Hercbergs A, Davis FB, Davis PJ (2008). Resveratrol is pro-apoptotic and thyroid hormone is anti-apoptotic in glioma cells: both actions are integrin and ERK mediated. Carcinogenesis.

[103] Lin HY, Shih A, Davis FB, Tang HY, Martino LJ, Bennett JA, Davis PJ (2002). Resveratrol induced serine phosphorylation of p53 causes apoptosis in a mutant p53 prostate cancer cell line. J Urol.

[104] Wu X, Xu Y, Zhu B, Liu Q, Yao Q, Zhao G (2018). Resveratrol induces apoptosis in SGC-7901 gastric cancer cells. Oncol Lett.

[105] Zhang S, Cao HJ, Davis FB, Tang HY, Davis PJ, Lin HY (2004). Oestrogen inhibits resveratrol-induced post-translational modification of p53 and apoptosis in breast cancer cells. Br J Cancer.

[106] Lin HY, Sun M, Tang HY, Simone TM, Wu YH, Grandis JR, Cao HJ, Davis PJ, Davis FB (2008). Resveratrol causes COX-2- and p53-dependent apoptosis in head and neck squamous cell cancer cells. J Cell Biochem.

[107] Liu Y, Tong L, Luo Y, Li X, Chen G, Wang Y (2018). Resveratrol inhibits the proliferation and induces the apoptosis in ovarian cancer cells via inhibiting glycolysis and targeting AMPK/mTOR signaling pathway. J Cell Biochem.

[108] Jia B, Zheng X, Wu ML, Tian XT, Song X, Liu YN, Li PN, Liu J (2021). Increased reactive oxygen species and distinct oxidative damage in resveratrol-suppressed glioblastoma cells. J Cancer.

[109] Wang G, Dai F, Yu K, Jia Z, Zhang A, Huang Q, Kang C, Jiang H, Pu P (2015). Resveratrol inhibits glioma cell growth via targeting oncogenic microRNAs and multiple signaling pathways. Int J Oncol.

[110] Öztürk Y, Günaydın C, Yalçın F, Nazıroğlu M, Braidy N (2019). Resveratrol Enhances Apoptotic and Oxidant Effects of Paclitaxel through TRPM2 Channel Activation in DBTRG Glioblastoma Cells. Oxid Med Cell Longev.

[111] Dörrie J, Gerauer H, Wachter Y, Zunino SJ (2001). Resveratrol induces extensive apoptosis by depolarizing mitochondrial membranes and activating caspase-9 in acute lymphoblastic leukemia cells. Can Res.

[112] Lin H, Xiong W, Zhang X, Liu B, Zhang W, Zhang Y, Cheng J, Huang H (2011). Notch-1 activation-dependent p53 restoration contributes to resveratrol-induced apoptosis in glioblastoma cells. Oncol Rep.

[113] Ashrafizadeh M, Taeb S, Haghi-Aminjan H, Afrashi S, Moloudi K, Musa AE, Najafi M, Farhood B (2021). Resveratrol as an Enhancer of Apoptosis in Cancer: A Mechanistic Review. Anticancer Agents Med Chem.

[114] Juan ME, Wenzel U, Daniel H, Planas JM (2008). Resveratrol induces apoptosis through ROS-dependent mitochondria pathway in HT-29 human colorectal carcinoma cells. J Agric Food Chem.

[115] Lee SC, Chan JY, Pervaiz S (2010). Spontaneous and 5-fluorouracil-induced centrosome amplification lowers the threshold to resveratrol-evoked apoptosis in colon cancer cells. Cancer Lett.

[116] Ramachandran L, Nair CKK (2011). Therapeutic potentials of silver nanoparticle complex of α-lipoic acid. Nanomater Nanotechnol.

[117] Vyas D, Laput G, Vyas AK (2014). Chemotherapy-enhanced inflammation may lead to the failure of therapy and metastasis. Onco Targets Ther.

[118] Haghi Aminjan H, Abtahi SR, Hazrati E, Chamanara M, Jalili M, Paknejad B (2019). Targeting of oxidative stress and inflammation through ROS/NF-kappaB pathway in phosphine-induced hepatotoxicity mitigation. Life Sci.

[119] Shayesteh MRH, Haghi-Aminjan H, Mousavi MJ, Momtaz S, Abdollahi M (2019). The protective mechanism of cannabidiol in cardiac injury: a systematic review of non-clinical studies. Curr Pharm Des.

[120] Moutabian H, Ghahramani-Asl R, Mortezazadeh T, Laripour R, Narmani A, Zamani H, Ataei G, Bagheri H, Farhood B, Sathyapalan T *et al*: The cardioprotective effects of nano-curcumin against doxorubicin-induced cardiotoxicity: A systematic review. *BioFactors (Oxford, England)* 2022. 10.1002/biof.1823 35080781

[121] Zhang Q, Wu L (2022). In vitro and in vivo cardioprotective effects of curcumin against doxorubicin-induced cardiotoxicity: a systematic review. J Oncol.

[122] Askari H, Rajani SF, Poorebrahim M, Haghi-Aminjan H, Raeis-Abdollahi E, Abdollahi M (2018). A glance at the therapeutic potential of irisin against diseases involving inflammation, oxidative stress, and apoptosis: an introductory review. Pharmacol Res.

[123] Sheikholeslami S, Khodaverdian S, Dorri-Giv M, Mohammad Hosseini S, Souri S, Abedi-Firouzjah R, Zamani H, Dastranj L, Farhood B (2021). The radioprotective effects of alpha-lipoic acid on radiotherapy-induced toxicities: a systematic review. Int Immunopharmacol.

[124] Farhood B, Mortezaee K, Goradel NH, Khanlarkhani N, Salehi E, Nashtaei MS, Najafi M, Sahebkar A (2019). Curcumin as an anti-inflammatory agent: Implications to radiotherapy and chemotherapy. J Cell Physiol.

[125] Jeong BK, Song JH, Jeong H, Choi HS, Jung JH, Hahm JR, Woo SH, Jung MH, Choi BH, Kim JH (2016). Effect of alpha-lipoic acid on radiation-induced small intestine injury in mice. Oncotarget.

[126] Raj V, Bhadauria AS, Singh AK, Kumar U, Rai A, Keshari AK, Kumar P, Kumar D, Maity B, Nath S (2019). Novel 1,3,4-thiadiazoles inhibit colorectal cancer via blockade of IL-6/COX-2 mediated JAK2/STAT3 signals as evidenced through data-based mathematical modeling. Cytokine.

[127] Hashemi Goradel N, Najafi M, Salehi E, Farhood B, Mortezaee K (2019). Cyclooxygenase-2 in cancer: A review. J Cell Physiol.

[128] Bowman T, Garcia R, Turkson J, Jove R (2000). STATs in oncogenesis. Oncogene.

[129] Bromberg JF (2001). Activation of STAT proteins and growth control. BioEssays.

[130] Bromberg J (2002). Stat proteins and oncogenesis. J Clin Investig.

[131] Epling-Burnette PK, Liu JH, Catlett-Falcone R, Turkson J, Oshiro M, Kothapalli R, Li Y, Wang JM, Yang-Yen HF, Karras J (2001). Inhibition of STAT3 signaling leads to apoptosis of leukemic large granular lymphocytes and decreased Mcl-1 expression. J Clin Investig.

[132] Yu H, Jove R (2004). The STATs of cancer–new molecular targets come of age. Nat Rev Cancer.

[133] Barker HE, Paget JT, Khan AA, Harrington KJ (2015). The tumour microenvironment after radiotherapy: mechanisms of resistance and recurrence. Nat Rev Cancer.

